# Plasma and Ocular Prednisolone Disposition after Oral Treatment in Cats

**DOI:** 10.1155/2013/209439

**Published:** 2013-08-29

**Authors:** María J. Del Sole, Paula Schaiquevich, Marcelo A. Aba, Carlos E. Lanusse, Laura Moreno

**Affiliations:** ^1^Hospital de Pequeños Animales, CIVETAN-CONICET, Facultad de Ciencias Veterinarias (UNCPBA), Campus Universitario, Paraje Arroyo Seco s/n, 7000 Tandil, Buenos Aires, Argentina; ^2^Laboratorio de Farmacología, CIVETAN-CONICET, Facultad de Ciencias Veterinarias (UNCPBA), 7000 Tandil, Argentina; ^3^Unidad de Farmacocinética Clínica, CONICET, Hospital de Pediatría JP Garrahan, 1245 Buenos Aires, Argentina; ^4^Laboratorio de Endocrinología, CIVETAN-CONICET, Facultad de Ciencias Veterinarias (UNCPBA), 7000 Tandil, Argentina

## Abstract

*Objective*. To evaluate the plasma and aqueous humor disposition of prednisolone after oral administration in cats. *Methods*. Six cats were administered with a single oral dose of prednisolone (10 mg). Blood and aqueous humor samples were serially collected after drug administration. Prednisolone concentrations in plasma and aqueous humor were measured at 0.25, 0.5, 1.0, 1.5, 2.0, 3.0, 4.0, and 5.0 h after administration by a high-performance liquid chromatographic analytical method developed and validated for this purpose. *Results*. Mean ± standard error (SE) of maximum plasma prednisolone concentration (300.8 ± 67.3 ng/mL) was reached at 1 h after administration. Prednisolone was distributed to the aqueous humor reaching a mean peak concentration of 100.9 ± 25.5 ng/mL at 1.25 h after administration. The mean ± SE systemic and aqueous humor exposure (AUC) was 553.3 ± 120.0 ng∗h/mL and 378.8 ± 64.9 ng∗h/mL, respectively. A high AUC_aqueous humor_/AUC_plasma_ ratio was observed (0.68 ± 0.13). The mean half-life time of elimination in plasma and aqueous humor was 0.87 ± 0.16 h and 2.25 ± 0.44 h, respectively. *Clinical Significance*. The observed high ratio between aqueous humor and plasma prednisolone concentrations indicates that extensive penetration of prednisolone to the anterior segment of the eye may occur. This is the first step that contributes to the optimization of the pharmacological therapeutics for the clinical treatment of uveitis.

## 1. Introduction

Uveitis is a frequent ophthalmic disorder in domestic cats commonly associated with perforating or blunt traumas, systemic diseases, neoplasia, intraocular surgery, and lens-induced uveitis [1]. Currently, the treatment of feline uveitis of any etiology is based on topical and/or systemic corticosteroid or anti-inflammatory nonsteroidal agents [1, 2]. Despite the fact that the use of potent anti-inflammatory drugs carries the risk of side effects, corticosteroids remain the mainstay of the prevention and treatment of many painful and potentially blinding ocular diseases in different species [3]. Prednisolone is a synthetic adrenocortical steroid used primarily due to its anti-inflammatory activity in several diseases [4]. In addition, due to the potential antiangiogenic, antiedematous, antiapoptotic, and antiproliferative effects of prednisolone, this steroid gained wide use in the treatment of both anterior and posterior ocular segment diseases [5].

Although topical administration of prednisolone is widely used in the treatment of anterior uveitis, oral administration is also used in traumatic, after intraocular surgery, and neoplastic uveitis [1]. However, there is limited information about ocular prednisolone disposition after its oral administration in small species including the cat.

Previous studies on plasma pharmacokinetics of prednisolone have been performed in humans, rats, rabbits, cows, sheep, horses, dogs, and cats [6–13], but there are no reports about ocular disposition of the corticoid after oral administration in any of those species. In order to characterize the pharmacokinetics of prednisolone in different biological matrices, an analytical technique needs to be developed to obtain precise and accurate results. Different techniques were previously reported in the literature to estimate steroids concentrations in biological samples [14–20]. However, the methodologies to measure prednisolone in aqueous humor are limited, and there are no previous reports about a specific high-performance liquid chromatographic (HPLC) analytical method to quantify prednisolone in plasma and/or aqueous humor of cats.

Thus, the main goal of the current work was to evaluate the systemic kinetic behaviour of prednisolone and its distribution to aqueous humor after oral administration to cats. The work included the development and validation of an analytical HPLC methodology to quantify prednisolone in cat plasma and aqueous humor.

## 2. Materials and Methods

### 2.1. Animals

Six intact young European short-hair male cats with an average ± standard deviation (SD) weight of 4.2 ± 0.5 kg, obtained from the research colony of Facultad de Ciencias Veterinarias (UNCPBA), were used in the present study. Animals were housed individually in a temperature- and light-controlled environment (fluorescent lights were automatically turned on and off every 12 h) and fed with a balanced diet and water *ad libitum*. They were acclimated to human contact for 4 to 6 weeks prior to the study. Previous to their inclusion in the study it was assured that the animals were in healthy physical and ocular condition, throughout physical and ocular examination. Ocular examination included Schirmer tear test measurements (Schirmer tear test strips, Schering-Plough Animal Health Corp., Union, New Jersey, USA), fluorescein staining (Love Sudamericana Laboratory, Argentina), applanation tonometry (Tono-Pen XL, Mentor, Norwell, MA, USA), slit lamp biomicroscopy (HLS 150, Heine Optotechnik, Herrsching, Germany), direct (Heine Beta 200, Heine Optotechnik, Herrsching, Germany) and indirect (20-D lens, Ocular Instruments, Bellevue, Wash; Heine Omega 150, Heine Optotechnik, Herrsching, Germany) ophthalmoscopies. During the sampling period and the following 24 h, the animals were evaluated for aqueous flare and others signs of uveitis by slit lamp biomicroscopy. The cats did not receive any medications containing prednisone or prednisolone prior to the study (including topical preparations). All animal procedures were conducted in strict accordance with the Association for Research in Vision and Ophthalmology Statement for the Use of Animals in Ophthalmic and Vision Research and Animal Welfare Policy (Act 087/02, Facultad de Ciencias Veterinarias, UNCPBA, http://www.vet.unicen.edu.ar).

### 2.2. Experimental Administration and Sample Collection

Immediately after oral administration of 10 mg of prednisolone (Prednisolona Syntex, Syntex S.A., Argentina), cats were anesthetized using I.M. ketamine (7 mg/kg)-xylazine (1 mg/kg) and maintained with I.V. ketamine (3.5 mg/kg)-xylazine (0.1 mg/kg) administered 5 minutes previous to each sample extraction. Blood samples (2 mL) were taken through preplaced cephalic antebrachial intravenous catheters before and at 0.25, 0.5, 1.0, 1.5, 2.0, 3.0, 4.0, and 5.0 h after prednisolone administration. Blood samples were centrifuged at 2000 ×g for 15 min, and the separated plasma was transferred to plastic tubes. Aqueous humor samples were obtained alternating between each eye by paracentesis of the anterior chamber at the same blood sampling times. Aqueous humor samples (450 *μ*L) were collected slowly using a disposable 1 mL syringe with a 25 gauge needle, with the minimal vacuum needed to obtain the samples, as previously described [21]. Both plasma and aqueous humor samples were stored at −20°C until HPLC analysis.

### 2.3. Sample Preparation

All plasma and aqueous humor samples were vortexed, and aliquots of 300 *μ*L of plasma or 450 *μ*L of aqueous humor were placed into glass tubes. Plasma and aqueous humor samples were spiked with 40 *μ*L and 45 *μ*L of the internal standard (dexamethasone 1 *μ*g/mL), respectively. Prednisolone was extracted by the addition of 1 mL of ethyl acetate under a high speed vortexing shaker over 10 min. After centrifugation at 2500 ×g for 10 min (4°C) to allow phase separation, the clear supernatant was transferred to a 5 mL glass tube. This volume was evaporated (45°C) to dryness in a vacuum concentrator and then reconstituted with 300 *μ*L of mobile phase.

### 2.4. HPLC Analysis

Experimental and fortified plasma and aqueous humor samples were analysed for prednisolone by HPLC. After extraction, 100 *μ*L aliquots were injected in a Shimadzu Chromatography system (Shimadzu Corporation, Kyoto, Japan). The equipment was composed of two LC-10AS solvent pumps, an automatic sample injector (SIL-10A) with a 100 *μ*L loop, an ultraviolet visible spectrophotometer detector (UV) (SPD-10A), a column oven (Eppendorf TC-45, Eppendorf, Madison, WI, USA) set at 30°C, and a CBM-10A data integrator. Data and chromatograms were collected and analysed using the Class LC10 software (SPD-10A, Shimadzu Corporation, Kyoto, Japan). A C18 reversed-phase column (Kromasil, Eka Chemicals AB, NY, USA) of 250 mm × 4.6 mm with 5 *μ*m particle size was used for separation. Elution from the stationary phase was carried out at a flow rate of 1.2 mL/min using acetonitrile and water (36 : 64) in isocratic mode as mobile phase during 11 min. The detector was set at 254 nm. A complete validation of the analytical procedures for the extraction and quantification of prednisolone in plasma and aqueous humor was performed before starting the analysis of the biological samples. Recovery of prednisolone was estimated by the comparison of the peak areas from spiked plasma and aqueous humor samples with the areas resulting from direct injections of standards in mobile phase. Precision (intra- and inter-assay) was determined by analyzing replicates of each matrix sample enriched with prednisolone standard to achieve 50, 100 and 200 ng/mL for plasma and 10, 50 and 100 ng/mL for aqueous humor. The intraday and interday precision and accuracy of the method were evaluated by coefficient of variation (CV) and relative error (RE; RE = 100 × [(predicted concentration − nominal concentration)/nominal concentration]). The theoretical limit of quantification (LOQ) for prednisolone in plasma and aqueous humor were determined comparing the signal of zero samples of the different biological matrices fortified with the internal standard and measurement of the baseline noise at the retention time of prednisolone. The mean baseline noise plus six standard deviations was defined as the theoretical LOQ. The robustness was evaluated on the retention time (*t*_*R*_), recovery and repeatability of the method (% CV) by deliberate variations of the column temperature (30 and 35°C), flow rate (1 and 1.2 mL/min), and brand columns (Kromasil, Eka Chemicals AB, NY, USA and Phenomenex, CA, USA).

### 2.5. Pharmacokinetic Analysis

The pharmacokinetic analysis of prednisolone after oral administration to cats was carried out using the program PK Solution (Summit Research Services, Ashland, USA). Pharmacokinetic analysis of the experimental data was performed by a noncompartmental analysis. The following equation [22] was used to describe the biexponential concentration versus time curves for prednisolone in plasma and aqueous humor for each cat after the oral administration: *C*_*p*_ = *Be* ^−*βt*^ − *Ae* ^−*at*^, where *C*_*p*_ = concentration (ng/mL) in plasma or aqueous humor at time *t* after administration, *A* is concentration at time zero extrapolated from the absorption phase (ng/mL), *B* is concentration at time zero extrapolated from the elimination phase (ng/mL), *e* is base of the natural logarithm, *β* is terminal slope (h^−1^), and *a* is the slope obtained by feathering which represents the first order absorption rate constant (*k*_abs_) (h^−1^). No lag time was observed for plasma or aqueous humor pharmacokinetics. The maximum concentration (*C*_max⁡_) was obtained from the observed data. The area under the concentration (AUC) versus time curves was calculated by the trapezoidal rule [23]. The absorption (*T*_1/2abs_) and the elimination (*T*_1/2el_) half-lives were calculated as  ln⁡ 2/*k*_abs_ and  ln⁡ 2/*β*, respectively. Statistical moment theory was applied to calculate the mean residence time (MRT) in plasma and aqueous humor as follows [23]: MRT = AUMC/AUC, where AUC was defined previously and AUMC is the area under the curve for the first moment, obtained by the product of time and the plasma or aqueous humor drug concentrations versus time from zero to infinity [24].

### 2.6. Statistical Data Analysis

The pharmacokinetic parameters are reported as mean ± SE and the concentration data as mean ± SD. Student's *t*-test was used to compare parameters. A value of *P* < 0.05 was considered statistically significant.

## 3. Results

Prednisolone plasma and aqueous humor concentrations after oral administration of 10 mg of prednisolone to cats were quantified using the analytical assay developed in the present work. The calibration curves for prednisolone were linear in the range of 25–500 and 5–200 ng/mL (*r* ^2^ > 0.993 and 0.999) for plasma and aqueous humor, respectively. The linear regression was further supported by a homogeneous distribution of residuals and homoscedasticity of the variance. The theoretical LOQ of the method was 25 and 5 ng/mL for plasma and aqueous humor, respectively. The method exhibited a high degree of intraday and interday precision and accuracy as demonstrated by low CV and RE. The values of the main parameters determined in the validation methodology are shown in Table 1. Deliberate variation of the method conditions had no significant effect on assay data or on chromatographic performance, indicating the robustness of method and its suitability for routine use and transference to other laboratories.

**Figure 1:**
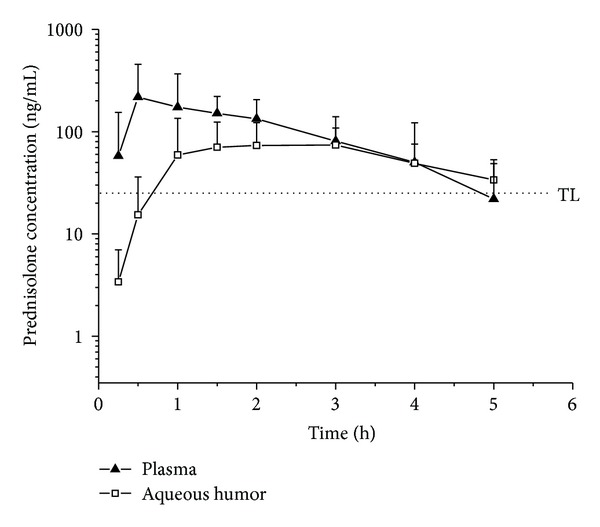
Mean ± SD prednisolone plasma and aqueous humor concentration profiles versus time obtained after its oral administration (10 mg) to cats. TL: therapeutic level concentration is reported as sufficient to suppress inflammation and minimise side effects in humans (25 ng mL^−1^).

Prednisolone concentrations were detected in both plasma and aqueous humor in all sampling periods after oral administration of 10 mg per cats. Mean plasma and aqueous humor concentration profiles versus time are shown in Figure 1. Prednisolone was detectable in plasma from 0.25 up to 5 h after administration. The mean ± SD plasma concentration at the first sampling time was 58.0 ± 96.8 ng/mL and reached a mean ± SE maximum (*C*_max⁡_) level of 300.8 ± 67.3 ng/mL at 1 ± 0.25 h (*T*_max⁡_, ±SE). In addition, the lowest mean ± SD plasma concentration of 22.0 ± 26.6 ng/mL was observed after 5 hours of drug administration. In aqueous humor, the concentration measured at 0.25 h after administration was lower than the LOQ, and the mean *C*_max⁡_ was 100.9 ± 25.5 ng/mL at 1.25 ± 0.30 h (*T*_max⁡_). Prednisolone concentration was higher in plasma than in aqueous humor up to 2 hours after drug administration, and thereafter both concentrations were similar as shown in Figure 1. At the last sampling time (5 h), the concentration in aqueous humor was higher than that observed in plasma. Plasma and aqueous humor calculated pharmacokinetic parameters are summarized in Table 2. The prednisolone absorption process to the systemic circulation was fast with a mean absorption half-life (*T*_1/2abs_, ±SE) of 0.37 ± 0.07 h. The mean half-life time of elimination (*T*_1/2el_) in plasma was 0.87 ± 0.16 h, and meanwhile the prednisolone *T*_1/2el_ in the aqueous humor was longer (2.25 ± 0.44 h). Prednisolone plasma and aqueous humor exposure, represented by the AUC ± SE, was 553.3 ± 120.0 ng∗h/mL and 378.8 ± 64.9 ng∗h/mL, respectively. The mean ± SE AUC_aqueous humor_/AUC_plasma_ ratio was 0.68 ± 0.13.

## 4. Discussion

In the present study, we developed an analytical assay for quantification of prednisolone in aqueous humor and plasma and characterized prednisolone pharmacokinetics in plasma and aqueous humor after oral administration to cats. This is the first study to evaluate the disposition of prednisolone in aqueous humor after oral administration.

After systemic administration, once absorbed, drug molecules are distributed throughout the body in the circulating blood, and they must diffuse into the target tissues to exert the pharmacological effect. The concentration attained at the desired target tissue depends on the delivery process of the drug to the tissues and the ability of the drug to penetrate capillary endothelium and diffuse across cell membranes [25], where factors such as lipophilicity and the binding of the drug to plasma proteins and other tissue components play a critical role [26]. Therefore, the tissue distribution process varies widely for different drug molecules and has to be studied in each case [27].

After oral prednisolone administration to cats, we observed that plasma and aqueous humor concentrations were quantifiable at 0.25 h, demonstrating that both prednisolone absorption and aqueous humor distribution processes were fast. In addition, it has been previously reported that a prednisolone concentration of 25 ng/mL has to be attained in human aqueous humor in order to suppress inflammation [28]. Currently, there is no information about the aqueous humor level that needs to be attained to obtain an anti-inflammatory response in cats. Apart from this limitation, if we considered the level reported in humans, we could expect that the prednisolone aqueous humor concentrations attained in the present work should be pharmacologically active for at least 5 hours after the oral administration of 10 mg of prednisolone in cats (Figure 1).

In the current work, the maximal concentration of prednisolone (*C*_max⁡_) in plasma was attained at 1 h and reached a mean ± SE value of 300.8 ± 67.3 ng/mL, and the mean prednisolone plasma exposure, represented by the AUC, was 553.3 ng∗h/mL. While higher systemic exposure of prednisolone was previously reported in cats [13], the rate of drug absorption and elimination was comparable to our present results. A feasible explanation for the different results could be attributed to differences in animal weight and, thus, in volume of distribution. Besides, plasma *C*_max⁡_ values that were obtained after 10 mg oral prednisolone in rats (863 ng/mL) were higher than the values observed in the present experiment in cats. This is probably due to the lower volume of distribution of the rat [7]. 

In the present study, we reported plasma half-life time of elimination of 0.66 h, comparable to previous reports in cats [13]. On the contrary, longer *T*_1/2el_ values (around 3 h) were reported after oral prednisolone in humans [6, 29]. When corrected for dose and considering the volume of distribution, the systemic exposure in human (AUC) is higher than that in cats probably because the 3-fold difference in *T*_1/2el_ between species. The lower plasma *C*_max⁡_ and AUC and the shorter prednisolone *T*_1/2el_ in cats with respect to humans may be associated with a higher metabolism and/or elimination rate or a less protein-bound prednisolone (transcortin) in this species. With important interspecies variations, prednisolone protein binding may affect pharmacokinetic parameters such as drug concentration, volume of distribution, and elimination rate which could explain the differences in distribution process and volume between cats and humans [30].

The aqueous humor drug exposure to prednisolone was lower than that calculated for plasma (AUC = 553.3 ± 120 ng∗h/mL) leading to an AUC_aqueous  humor_/AUC_plasma_ ratio of 0.68 ± 0.13, indicating an extensive prednisolone distribution into the aqueous humor after systemic administration to cats. Interestingly, aqueous humor *T*_1/2el_ and mean residence time were much longer than those observed in plasma (*P* < 0.01), indicating a longer residence in the eye.

According to the results previously reported for dexamethasone, another corticosteroid widely used for uveitis treatment, after a single oral dose of 7.5 mg the drug penetrates into the posterior segment in humans and the ratio mean *C*_max⁡ vitreous_/*C*_max⁡ plasma_ was approximately equal to 0.084. Assuming that this ratio is comparable between dexamethasone and prednisolone and similar between humans and cats, based on the plasma *C*_max⁡_ obtained in the present study (300.8 ± 67.3 ng/mL), the vitreous levels should be about 25 ng/mL. Hence, we suggest that a therapeutic drug level would be attained in the vitreous of our animal model after 10 mg of prednisolone [31]. If we considered this ratio, we can suppose that the vitreous humor level after oral administration of prednisolone should be lower than that in aqueous humor. Thus, assuming that the rate constant of drug transfer from the plasma to the vitreous is comparable between humans and cats, and that the therapeutic level in cats is similar to that in humans (25 ng/mL) [28], then based on the plasma *C*_max⁡_ obtained in the present study (300.8 ± 67.3 ng/mL) we could hypothesize that a therapeutic drug level would be attained in the vitreous of our animal model after 10 mg of prednisolone. However, the interval of time during which prednisolone vitreous concentrations are higher than 25 ng/mL has to be determined.

Anaesthesia for short periods was necessary to obtain the aqueous humor samples. For this purpose, similar to previous works, the animals were administered at each sampling time with ketamine and xylazine, since no interaction between prednisolone and these anaesthetic drugs has been observed [32, 33]. As described previously, under our experimental conditions, a single paracentesis does not induce ocular inflammation in cats [21]. Although four repetitive paracenteses were performed in this study, with a minimal interval of 45 minutes and a maximal of 120 minutes between samples, we did not observe any clinical signs of blood ocular barriers (BOB) breakdown during the sampling period or during the next 24 h, when the cats were examined before reposition to the research colony. It could be possible that the prednisolone directly reduced PGE synthesis and increased vascular stability avoiding BOB breakdown [34]. However, subclinical BOB breakdown could have occurred during sampling in the present experiment, and this could have increased slightly the aqueous humor drug levels. To avoid this situation, a microdialysis sampling technique is being developed to be used in future studies of prednisolone disposition in the eye.

Although the low content of aqueous humor proteins could result in a greater fraction of free prednisolone with respect to the plasma content, the BOB subclinical breakdown may lead to increased transcortin levels over time compensating the physiologic low levels of proteins.

In conclusion, the simple, precise, and accurate method developed and validated to quantify prednisolone in plasma and aqueous humor allowed us to obtain novel pharmacology-based information on the distribution of prednisolone in cats. This is a useful first step to evaluate the potential of prednisolone as an anti-inflammatory systemic drug for use in feline anterior uveitis. The pharmacokinetic characterization of prednisolone in plasma and aqueous humor after oral administration to cats indicates that the drug penetrates into the anterior chamber of the eye. Follow-up studies to characterize the pattern of distribution in the vitreous humor and to determine the anti-inflammatory levels of prednisolone are required to further evaluate the potential of this drug as an anti-inflammatory drug in the treatment of uveitis.

## Figures and Tables

**Table 1:** HPLC analytical validation.

Chromatographic parameter	Plasma	Aqueous humor
Absolute recovery range (%)	72–78	89–93
Intraday precision (%CV)	3.2–5.5	3.3–4
Interday precision (%CV)	4.2–7.6	2.3–11.1
Interday accuracy (%RE)	5.1–13.1	7.0–17.2
LOD (ng/mL)	4.3	1.3
LOQ (ng/mL)	25	5
Precision LOQ (%CV)	12.4	5.8
Stability (%CV)	3.8–4.5	0.9–4.6

CV: coefficient of variation; LOD: limit of detection; LOQ: limit of quantification; RE (relative error) = 100 × [(predicted concentration − nominal concentration)/nominal concentration].

**Table 2:** Plasma and aqueous humor pharmacokinetic parameters for prednisolone after a single oral administration (10 mg) to cats.

Pharmacokinetic parameter	Plasma		Aqueous humor	
	Mean	SE	Mean	SE
*T*_1/2abs_ (h)	0.37	0.07	0.55	0.09
*C*_ max_ (ng/mL)	300.8	67.3	100.9	25.5
*T*_ max_ (h)	1	0.25	2.25	0.31
MRT (h)	2.24	0.42	4.39	0.64
*T*_1/2el_ (h)	0.87	0.16	2.25	0.44
AUC (ng·h/mL)	553.3	120.0	378.8	64.9

*T*_1/2abs_: absorption half-life; *C*_max_: maximum concentration; *T*_max_: time to maximum concentration; MRT: mean residence time; *T*_1/2el_: elimination half-life; SE: standard error; AUC: area under the concentration—time curve.
